# Sequencing and analysis of the complete mitochondrial genomes of *Toona sinensis* and *Toona ciliata* reveal evolutionary features of *Toona*

**DOI:** 10.1186/s12864-023-09150-6

**Published:** 2023-02-01

**Authors:** Youli Li, Min Gu, Xuanzhe Liu, Jianna Lin, Huier Jiang, Huiyun Song, Xingcui Xiao, Wei Zhou

**Affiliations:** 1 College of Forestry and Landscape Architecture, South China Agricultural University, Guangzhou, 51000 Guangdong China; 2 Sichuan Academy of Forestry Sciences, Chengdu, 61008 Sichuan China

**Keywords:** *Toona sinensis*, *Toona ciliate*, High-throughput sequencing, Mitochondria genome, Phylogenetic relationship

## Abstract

**Background:**

*Toona* is a critical genus in the Meliaceae, and the plants of this group are an asset for both restorative and restorative purposes, the most flexible of which are *Toona sinensis* and *Toona ciliata*. To concentrate on the advancement of mitochondrial(Mt) genome variety in *T.sinensis* and *T.ciliata*, the Mt genomes of the two species were sequenced in high throughput independently, after de novo assembly and annotation to construct a Mt genome map for comparison in genome structure. Find their repetitive sequences and analyze them in comparison with the chloroplast genome, along with Maximum-likelihood(ML) phylogenetic analysis with 16 other relatives.

**Results:**

(1) *T. sinensis* and *T.ciliata* are both circular structures with lengths of 683482 bp and 68300 bp, respectively. They share a high degree of similarity in encoding genes and have AT preferences. All of them have the largest Phe concentration and are the most frequently used codons. (2) Both of their Mt genome are highly preserved in terms of structural and functional genes, while the main variability is reflected in the length of tRNA, the number of genes, and the value of RSCU. (3) *T. siniensis* and *T. ciliata* were detected to have 94 and 87 SSRs, respectively, of which mononucleotides accounted for the absolute proportion. Besides, the vast majority of their SSRs were found to be poly-A or poly-T. (4)10 and 11 migrating fragments were identified in the comparison with the chloroplast genome, respectively. (5) In the ML evolutionary tree, *T.sinensis* and *T.ciliata* clustered individually into a small branch with 100% support, reflecting two species of *Toona* are very similarly related to each other.

**Conclusions:**

This research provides a basis for the exploitation of *T.sinensis* and *T.ciliata* in terms of medicinal, edible, and timber resources to avoid confusion; at the same time, it can explore the evolutionary relationship between the *Toona* and related species, which does not only have an important practical value, but also provides a theoretical basis for future hybrid breeding of forest trees, molecular markers, and evolutionary aspects of plants, which has great scientific significance.

**Supplementary Information:**

The online version contains supplementary material available at 10.1186/s12864-023-09150-6.

## Introduction

*Toona* plants have magnificent material, straight surface, and radiance, turning into the predominant furnishings and inside adornment wood, known as "Chinese mahogany", which is greatly esteemed by individuals [1–5]. *T.sinensis* and *T.ciliata* have the most noteworthy application in the *Toona*. *Toona sinensis (A. Juss) Roem* is a unique species of vegetable in China, its young shoots and leaves are crisp and juicy, fragrant and unique in flavor, it is a traditional and valuable woody vegetable that our people like to eat, and is also a local product for foreign trade export [6, 7]. *T.sinensis* is not only an excellent vegetable but also a natural green nutritious food of medicinal and food origin. It has somewhat high happiness of flavone and other pharmacologically dynamic mixtures [8, 9]. *T.ciliata* is a Grade II safeguarded plant, an important timber tree, and a therapeutic plant that has acquired broad consideration as of late [10–13]. The roots, stems, and leaves of *T.ciliata* can be utilized as medication and have successful restorative properties [14–16]. The monetary worth of this variety is quite high, and it is generally utilized and has extraordinary potential for advancement and utilization [17–20].

Mitochondria are organelles in higher plant cells with a semi-autonomous genetic system that provides the majority of the energy required for cellular and other life activities [21–24]. Mitochondria are particularly important in the study of the origin and evolution of living things. Mitochondrial DNA (mtDNA) is a genetic material found outside the nucleus that is normally a double-stranded circular molecule with a covalent closure [25–27]. Advanced plants have the largest mitochondria of any known higher organism species, ranging from 200 to 2400 kb [28–30].

Plant Mt genomes have been increasingly studied and more and more Mt genomes have been sequenced in recent years, which is very important for studying the diversity of biological phenotypes, functional diversity, as well as species evolution. This is critical for understanding biological phenological diversity, functional diversity, and the emergence of new functions during species evolution [31, 32].

Even though *Toona* plants have a long history of cultivation in China, most studies have been limited to chemical pathology, physiology, biochemistry, introduction, and breeding, with little research done on its origin, taxonomy, cytogenetics, and so on. There are still some issues with *Toona* classification, such as interspecific hybridization, that need to be addressed [33, 34]. Furthermore, *Toona* plants have a geographically dispersed distribution in China, resulting in a scarcity of natural forests and susceptibility to natural and anthropogenic breakage, *T.ciliata* has now been classified as an endangered species, listed as a Class II key protected wild plant in China, and included in the Reference List of Major Cultivated Precious Tree Species in China and [35–38].

Subsequently, this review, given Mt near genomic examination through trend-setting innovations, for example, sub-atomic sequencing of Mt DNA, makes it conceivable to concentrate on *Toona* further top to bottom according to a minuscule viewpoint notwithstanding plainly visible morphological characterization and makes the preservation of excellent hereditary assets of the imperiled species *T.ciliata*, determination and reproducing of good species, and advancement and usage with significant hypothetical and functional importance.

## Materials and methods

### Plant material, DNA extraction, and library construction

While *T.sinensis* was acquired from Pingxiang, Guangxi, *T.ciliata* was obtained from Baoshan, Yunnan (Longitude: 106.75 E, Latitude: 22.12 N.) Before this investigation, both species completed seedling trials and were found to be suitable for cultivation in Guangzhou, Guangdong. (Note: Professor Xiaoyang Chen and Teacher WeiZhou conducted a detailed identification of the plant material. The seed trial forest is situated near the South China Agricultural University's teaching and research facility in Guangzhou, China, at N23°16′ and E113°37′.)

High-quality total DNA is the primary prerequisite for obtaining the whole Mt genome sequence. Fresh leaves of *T.sinensis* and *T.ciliata* were taken and whole genome DNA was extracted by the CTAB [39] method. high-quality genome DNA was extracted and quality checked for purity, concentration, and integrity using Nanodrop [40], 1% (w/v) agarose gel electrophoresis. DNA samples that passed the electrophoresis test were randomly broken into fragments of approximately 350 bp in length using a Covaris ultrasonic fragmentation machine [41]. After processing, the DNA fragments were subjected to end repair, A-tail addition, sequencing junction addition, purification, PCR amplification, and other steps to complete the entire library preparation. After the library was constructed, the initial quantification was performed using Qubit 3.0, and the library was diluted to 2 ng/ul. The insert size (insert size) of the library was then detected using Agilent 2100 [42, 43]. After the inserts met the expectation, the effective concentration of the library was accurately quantified by Q-PCR [44] to ensure the quality of the library. After the libraries passed the test, they were sent to Guangzhou Ruike Gene Technology Co.

### Sequencing, assembly, and annotation

Qualified DNA libraries were sequenced using the Illumina HiSeq 4000 High-throughput Sequencing Platform. Once the sequencing was completed, the sequenced data were spliced into the Mt genome. The reads with low sequencing quality (< 40 bp in length) were filtered by Trimmomatic [45], the overlapping reads were filtered out by Blast to obtain Clean Data, and the sequencing data were analyzed by 15-mer using K-mer software to obtain high-quality reads. Assembly was performed using SOAP denovo [46] assembly software. The preliminary assembly results were optimized and holes were filled using krskgf and gapclose [47] software to obtain the specific assembly results.

The complete Mt genome sequence was annotated utilizing CPGAVAS [48] software together with DOGMA [49] software. Comparison analysis of blast on the proximal edge, followed by manual correction(Specific results of *T.sinensis* and *T.ciliata* annotations can be detailed in  Additional file 7 and 8: Appendices G and H, respectively). Transfer RNA (transfer RNA, tRNA) genes were identified along with manual correction employing tRNAscan-SE [50] software. The BLAST [51] search method was performed to align [52–54] and validate [55] the information sites such as gene boundaries, intron, exon, and coding regions.

The annotated genome sequences were submitted to NCBI according to the requirements, resulting in the definitive accession numbers *T.sinensis* (GenBank: OM574631.1) and *T.ciliata* (GenBank: OM574630.1).

### Superior Mt genome analysis

#### Structure and composition

Mitochondria were mapped by OGDRAW vl.2 (Organellar Genome DRAW) [56] online website (https://chlorobox.mpimp-golm.mpg.de/OGDraw.html). The circular structure of the genome sequence was mapped. The base content of the Mt genome was calculated using Editseq [57] software to obtain the ratio of A, T, C, G, and GC content respectively.

#### Frequency of codon usage

Considering the formula mentioned in Sharp PM literature [58], the utilization of relative equivalent codon use (RSCU) was examined utilizing CodonW [59] software.

### Simple sequence repeats

Simple Sequence Repeats (SSRs) of the Mt genome of T. sinensis and T. ciliata were analyzed using MISA [60] software, with the tandem repeat unit length and a minimum number of repeats set to > 10 for single nucleotide repeats, > 6 for dinucleotide repeats, and > 5 for trinucleotide, tetranucleotide, pentanucleotide, and hexanucleotide repeats. The minimum distance between SSRs was set to 100 bp.

### Chloroplast and Mt genomes

The chloroplast genome sequences of *T.sinensis* (GenBank: OK572965) and *T. ciliata* (GenBank: OK572964.1) on NCBI were uploaded by our group before completion. Match them with the mitochondrial genome for Blastn (https://blast.ncbi.nlm.nih.gov/Blast.cgi) to find out the migrating gene sequences. Regions with similarity greater than 90% and comparison lengths greater than 50 bp were screened as migration sequences.

### Phylogenetic tree analysis

Species (The specific Mt information etc. of the tree species can be detailed in a file called taxonomy in Additional file 4: Appendices D) with complete Mt genome arrangements and explanations in direct relation to the objective species were downloaded from NCBI for phylogenetic tree development. For more details on structural trees, the ML construction tree method is described in the folder titled "Description of the structure tree" in Additional file 4: Appendices D, while the Bayesian construction tree method is detailed in Additional file 5: Appendices E.

## Results

### Genome features

**Fig. 1:**
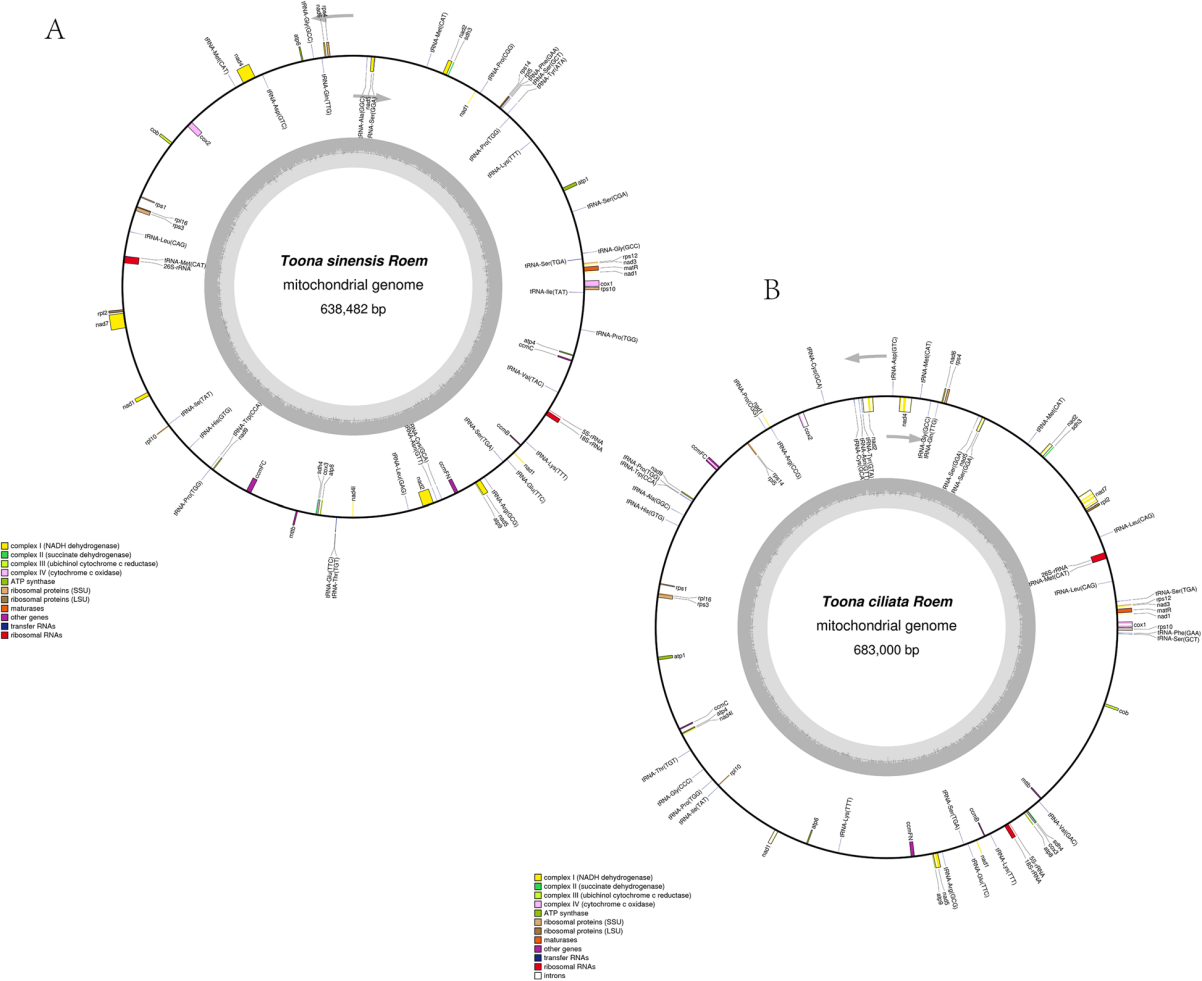
A map of the Mt genome of the Toona. **A** T.sinensis, **B** Toona ciliate. Reverse transcription is indicated by genes outside of the circles, and clockwise transcription is indicated by genes inside the circles. The two IR regions are represented by the thick black line on the outside circle. The GC content is represented by the inner nucleus' dark gray graph

The total Mt genome length of *T.ciliata* was 683,000 bp, the composition of bases was A (27.31%), T (27.29%), C (22.56%), and G (22.85%), and the C + G content was 45.40%. The size of the *T. sinensis* Mt genome was 638,482 bp, and its base makeup was A (27.35%), T (27.09%), C (22.79%), and G (22.76%), with a C + G content of 45.56%. All of them have a circumferential Mt genome construction, where their longest gene is the rrn26 gene in the transfer RNA, measuring 3116 bp (Table 1, Fig. 1).

**Table 1:** Results of mt DNA genome sequence analysis of two plants

	***Toona ciliata***		***Toona sinensis***	
**Type**	**Size**	**Proportion**	**Size**	**Proportion**
A content	186,538	27.31%	174,629	27.35%
T content	186,361	27.29%	172,983	27.09%
G content	156,039	22.85%	145,343	22.76%
C content	154,062	22.56%	145,527	22.79%
Total content	683,000		638,482	
G + C content	310,101	45.40%	290,870	45.56%
longest gene	3,116	50.77%	3,116	50.77%

Both mitochondria of the longest gene are 26S-rRNA, 50. The GC content of the gene is 50.77%

### Functional gene

#### Gene encoding protein

*T.ciliata* encodes 71 genes while *T.sinensis* encodes 72 genes. Protein-coding genes of both *Toona* plants are consistent in frequency, types, and measurements (Additional file 1: Appendices A), whereas the predominant divergence is in tRNAs, with *T.ciliata* encoding 33 tRNAs and *T. sinensis* encoding 34 (Additional file 2: Appendices B).

Employing NCBI-BLAST analysis, 38 genes encoding proteins were obtained on the Mt genomes of both *T.ciliata* and *T. sinensis*. We categorized the protein-encoding genes into the following eight categories according to their gene functions (Table 2): including Complex I genes ( *nad1, nad2, nad3, nad4, nad4L, nad5, nad6, nad7,* and *nad9*) involved in the synthesis of NADH deaminase subunits; Complex II genes ( *sdh3* and *sdh4*) participated in the synthesis of cytochrome b precursor subunits; Complex III gene (*cob*) implicated in the synthesis of the cytochrome C oxidase subunit; Complex IV genes (c*ox1, cox2,* and *cox3*); Complex V genes (*atp1, atp4, atp6, atp8,* and *atp9*), associated with the synthesis of ATP synthase subunits; Cytochrome c biosynthetic genes (*ccmB, ccmC, ccmFC* and *ccmFN*) engaged in the synthesis of cytochrome C synthase subunits; Ribosome protein genes synthesized by ribosome protein synthesis genes (*rps1, rps3, rps4, rps10, rps12, rpl2, rpl5, rpl10* and *rpl16*); The ribosomal RNA genes (*rrn5, rrn18* and rrn26) as well as the *matR* gene (encoding a maturation-like enzyme) and the *mttB* gene (encoding a transporter).

**Table 2:** Number and proportion of each type of SSR in *T.sinensis* and *T.ciliata*

	*Toona_ciliata*		*Toona_sinensis*	
Type	Number	Ration	Number	Ration
mono-nucleotides	75	78.13%	70	78.65%
di-nucleotides	14	14.58%	13	14.61%
tri-nucleotides	6	6.25%	6	6.74%
tetra-nucleotides	0	0.00%	1	1.12%
penta-nucleotides	0	0.00%	0	0.00%
hexa-nucleotides	1	1.04%	0	0.00%

#### Gene encoding tRNA

Utilizing tRNAscan-SE, 33 and 34 genes encoding transfer RNAs were identified separately on the Mt genomes of T.ciliata and T. sinensis.

In the *T.ciliata*, a total of 33 tRNAs encode 20 amino acids ranging from 66 bp-88 bp in length. five of these tRNASers, Leucine(LeU), Glycine(Gly), Gly, Cysteine(Cys), Argnine(Arg), and Lysine(Lys) each have two tRNAs encoding, Met and Pro are distributed with three tRNAs encoding, and the remaining amino acids all have one tRNA Editor. In contrast to *T.ciliata, T. sinensis* has 34 tRNAs encoding 20 amino acids, ranging from 63–167 bp in length. compared to *T.ciliata, T. sinensis* has 2 fewer tRNAs encoding Cys and Arg, but 3 more tRNAs encoding Proline(Pro), Isoleucine(ILe), and Glutamic acid(Glu) (Additional file 2: Appendices B).

### Codon Usage bias

RSCU (Relative Synonymous Codon Usage) is a relative synonymous codon usage measure, indicating the proportion of a given synonymous codon usage among all synonymous codons. The Mt genomes of *T.ciliata* and *T. sinensis* have a codon usage bias for all amino acids except for the Tryptophane (Trp) of only one codon, TGG.

The codon TTT was the most frequently accessed codon in the Mt protein-coding genes of *T.ciliata* and *T. sinensis*, with the second commonest codon being ATT and the third being TTC. The termination codon TAG was the least frequently addressed codon, being exclusively indexed on six and five occasions respectively (Additional file 3: Appendices C).

### SSRs

A total of 94 simple sequence repeats were detected in the mitochondrial genome of *T.sinensis* while 87 were detected in *T. ciliata* (Annex E). The distribution of each type of SSRs can be observed from the statistical results (Table 2), where *T. sinensis* mono-, di-, tri-, and hexa-nucleotides had 75, 14, 6, and 1, respectively. No tetra-nucleotides were detected and penta-nucleotides. *T.ciliata*, on the other hand, had 70, 13, 6, and 1, respectively. However, *T.ciliata* also detected 1 tetra-nucleotides (CGA).

The major repeat types of SSRs are single nucleotide repeats, with the number of A/T in the relevant single nucleotide repeats being much larger than the number of G/C (Table 3). The proportion of A/T on polynucleotide repeats is also greater than the proportion of G/C, judging from the data in Additional file 6: Appendices F. It is consistent with the results of their codon preferences.

**Table 3:** Distribution of the number of single nucleotide repeats

	***Toona_ciliata***		***Toona_sinensis***	
p1Type	**Number**	**Ration**	**Number**	**Ration**
A	**353**	**43.53%**	**301**	**40.35%**
T	**347**	**42.79%**	**374**	**50.13%**
G	**79**	**9.74%**	**71**	**9.52%**
C	**32**	**3.95%**	**0**	**0.00%**

### Genome alignment and migration sequence

Even though *T. sinensis* and *T.ciliata* mitochondrial genomes are up to four times longer than those of chloroplasts, they only have half as many protein-coding genes, making up less than one-fifth of the total length, whereas the proportion of protein-coding in chloroplasts is around 50% of the total length (Table 4). There were no introns found in the chloroplast genome of *T. sinensis* or *T. ciliata*, 21 introns were released in the mitochondrial genome, and the rRNA numbers of the two tree species were very congruent in both genomes. *T. sinensis* was larger than T.ciliata in the mitochondrial genome but had one more tRNA, which may be related to the exchange of genetic material in nuclear genes or cytoplasm. Both *T. sinensis* and *T. ciliata* had 37 numbers in the chloroplast genome.

**Table 4:** Comparison of chloroplast and mitochondrial genomes of *T. sinensis* and *T. ciliata*

	**T.ciliata**		**T.sinensis**	
	**Chloroplast**	**Mitochondrion**	**Chloroplast**	**Mitochondrion**
**Genome size(bp)**	**159618**	**683000**	**159139**	**638,482**
**GC(%)**	**37.89**	**45.4**	**37.9**	**45.56**
**Depth(X)**	**1,175**	**233**	**1,917**	**391**
**Genne no**	**132**	**71**	**132**	**72**
**Protein-coding sequence(%)**	**49.63**	**4.65**	**49.78**	**4.97**
**Intron no**	**not detected**	**21**	**not detected**	**21**
**tRNA no**	**37**	**33**	**37**	**34**
**tRNA Sequence(%)**	**1.76**	**0.37**	**1.77**	**0.41**
**rRNA no**	**8**	**3**	**8**	**3**
**rRNA Sequence(%)**	**5.67**	**0.76**	**5.69**	**0.81**

We discovered that *T. ciliata* had 11 migratory sequences and *T. sinensis* had 10 when we compared the chloroplast and mitochondrial genome sequences under the screening criteria of areas with similarity greater than 90% and comparison length greater than 50 bp(Table 5). The largest of these migratory sequence segments measured 4124 bp. Comparatively, we discovered that *T.sinensis* While only one sequence fragment of *T.ciliata* was consistent, with a variation of 1–8 bp, we discovered that only three sequence fragments of *T. sinensis* and mitochondria were consistent in size, with the others varying by 1–3 bp. The mitochondrial genome's recombination and gene rearrangement were linked to variations in sequence, which may indicate that after migratory integration, these fragments may have undergone separate replication and recombination within the mitochondrial genome recombination.

**Table 5:** Gene sequences of T. sinensis and T. ciliata mitochondrial genomes derived from the chloroplast genome

Species	Identity	Map length	Chloroplast start position	Chloroplast end position	Mitochondrial start position	Mitochondrial end position
***Toona sinensis*** **(CP ID:OK572965.1 Mt ID:NC_065061.1)**	**97.468**	**79**	**1**	**79**	**398,284**	**398,362**
	**95.077**	**2458**	**43876**	**46292**	**156,746**	**154,329**
	**96.392**	**1774**	**46606**	**48355**	**154,342**	**152,577**
	**93.671**	**79**	**54778**	**54856**	**213,727**	**213,805**
	**98.719**	**4137**	**87332**	**91456**	**237,563**	**233,440**
	**99.558**	**906**	**106599**	**107502**	**144,514**	**145,419**
	**98.516**	**1415**	**108259**	**109668**	**382,302**	**383,708**
	**93.939**	**66**	**110916**	**110981**	**131,432**	**131,497**
	**97.468**	**79**	**112237**	**112314**	**516,703**	**516,781**
	**98.516**	**1415**	**136179**	**137588**	**635,165**	**633,759**
***Toona ciliata*** **(CP ID:OK572964.1 Mt ID:NC_065060.1)**	**97.5**	**80**	**3**	**82**	**292,191**	**292,112**
	**99.642**	**279**	**41,482**	**41,759**	**333,893**	**333,615**
	**98.14**	**484**	**42,343**	**42,826**	**333,143**	**333,625**
	**95.556**	**2453**	**44,221**	**46,632**	**129,322**	**126,895**
	**97.463**	**1774**	**46,946**	**48,713**	**126,908**	**125,143**
	**93.671**	**79**	**55,119**	**55,197**	**154,865**	**154,787**
	**98.598**	**4136**	**87,716**	**91,840**	**210,015**	**205,899**
	**93.939**	**66**	**111,301**	**111,366**	**104,004**	**104,069**
	**97.468**	**79**	**112,623**	**112,700**	**183,954**	**184,032**
	**99.011**	**1415**	**136,667**	**138,076**	**424,683**	**426,096**
	**98.896**	**906**	**138,833**	**139,736**	**117,993**	**117,088**

### Phylogeny analysis

**Fig. 2:**
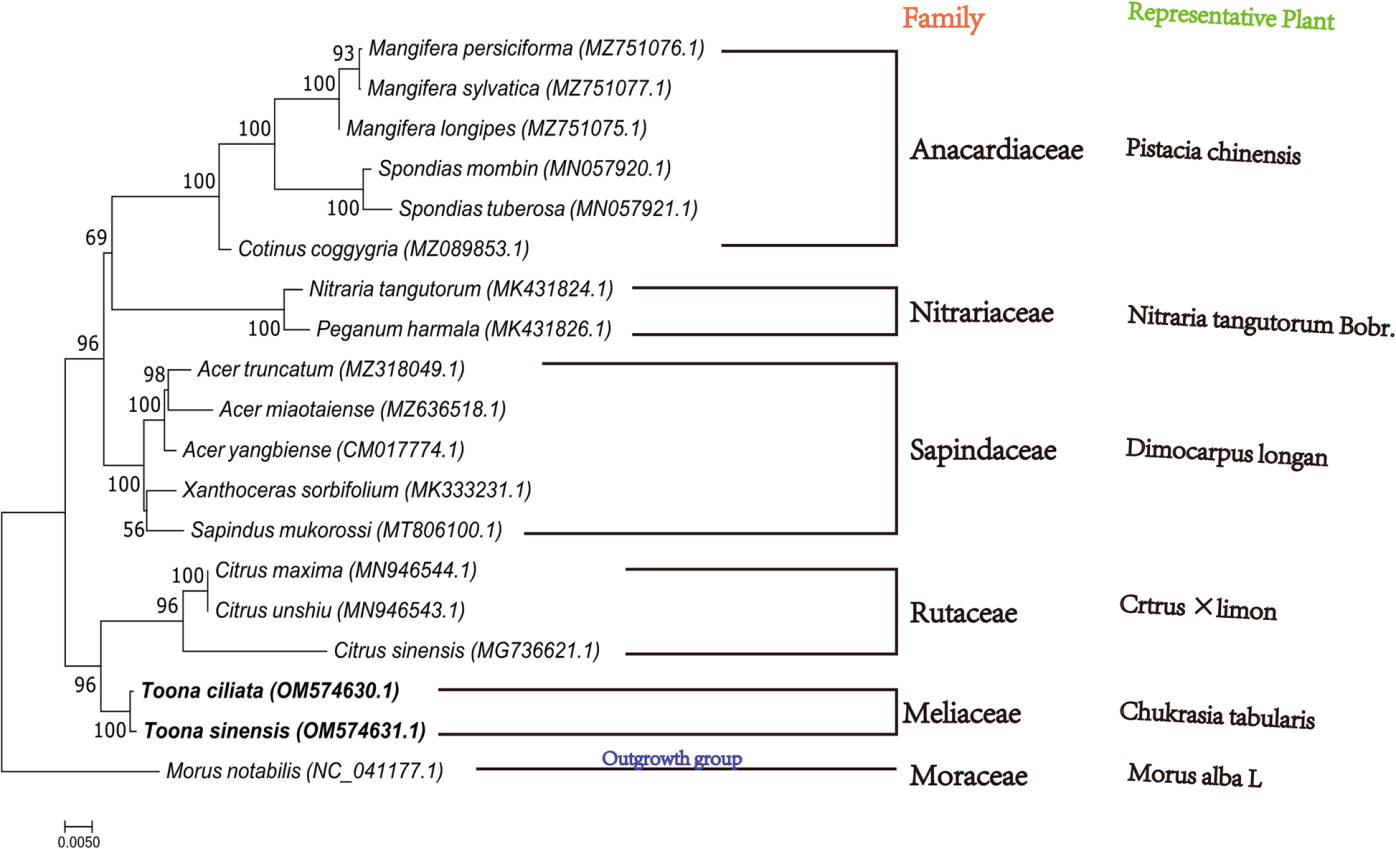
Phylogenetic trees constructed based on the ML method for 19 related plants

**Fig. 3:**
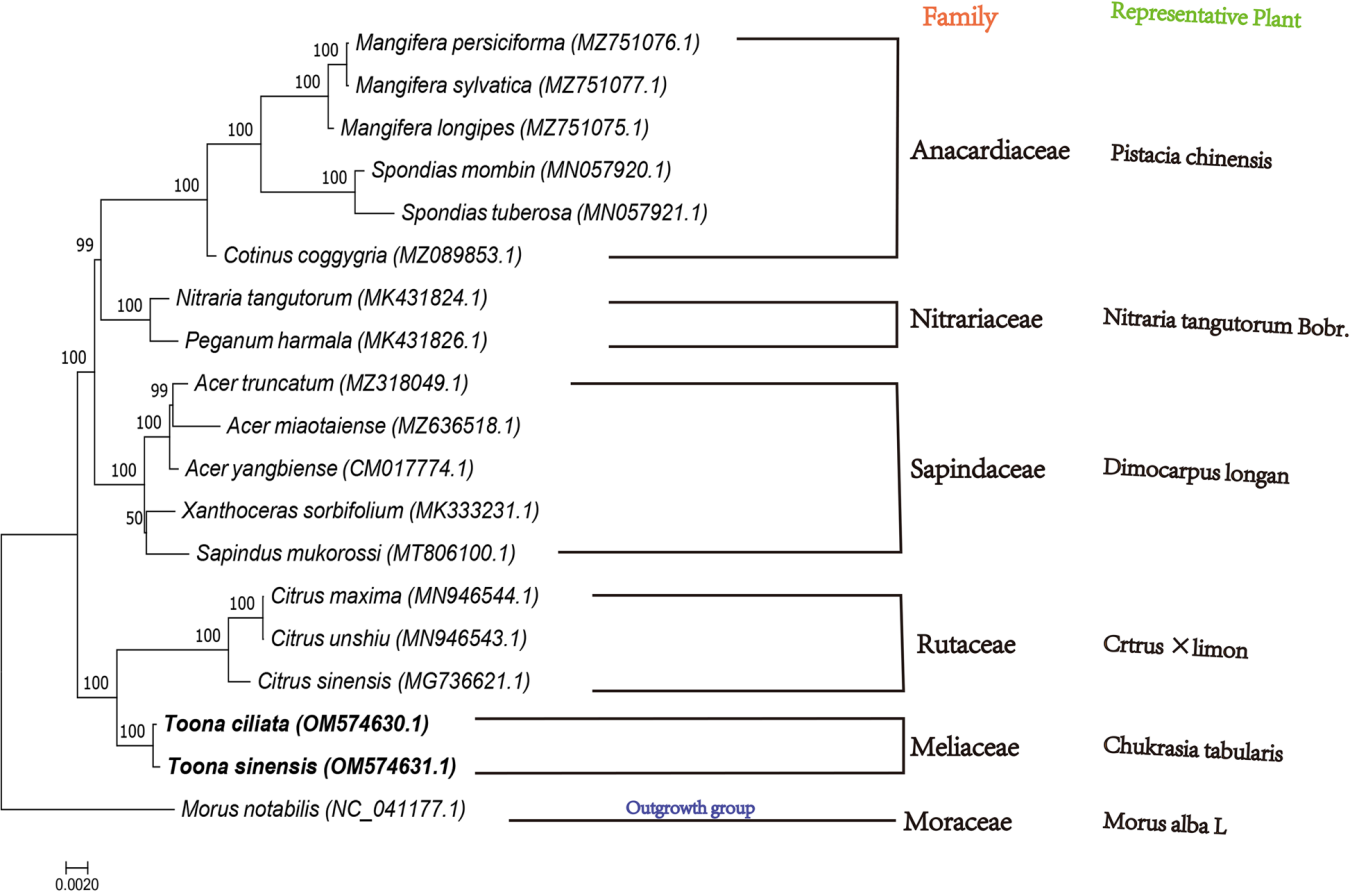
Phylogenetic trees constructed based on the BI method for 19 related plants

Aiming to ascertain the evolutionary status of *T.ciliata* and *T. sinensis* in the plant system, we downloaded the mtDNA sequences of the same ORDER relatives that have published their mtDNA sequences on NCBI. The two approaches of amino acid construction tree and DNA sequence construction tree are described in Additional file 3: Appendices C. Six Anacardiaceae species, five Sapindaceae species, three Rutaceae species, two Nitrariaceae species, and two Meliaceae species, *T.ciliata* (OM574630) and *T.sinensis* (OM574631), for a total of 18 tree species. The outgroup for the Mt genome was *Morus notabilis* (NC 041177.1), and an evolutionary tree was constructed using the maximum likelihood method using the software MEGA 11. Bayesian tree (BI) and maximum likelihood method (ML) to create phylogenetic tree topology are similar, only the support at a few branches varies(Only the values between the large branches clustered into Anacardiaceae and Nitrariaceae had large divergences, where the ML tree had a support of 69, while the BI tree was 99). In this research, the tree with the maximum likelihood tree is selected, detailed in Fig. 2, while the result regarding the BI development tree is detailed in Fig. 3. In the likelihood ML phylogenetic tree (Fig. 2), a total of 15 nodes were formed, nine of which had 100 percent support, except for the large branch of Anacardiaceae and Nitrariaceae, which had 69 percent support, and *Xanthoceras sorbifolium* (MK333231.1) and *Sapindus mukorossi* (MT806100.1), which formed a minor branch with only 56 percent support, but all the other nodes had no less than 93% support.

## Discussion

### Mitochondrial Structure and Genetic Information

In terms of GC content, gene content, and genetic codon usage preference, functional gene and the numerical Mt genomes of T.ciliata and T. sinensis were well conserved. Moreover, they are similar in the results of codon preference and RSCU values. These encoded genes are mainly concerned with the synthesis of ATP synthase subunits, cytochrome C synthesis, and ribosomal protein synthesis. This provides the theoretical conditions for the exploration of the mechanisms and pathways of metabolite synthesis reactions including respiration and other related metabolites between the two species.

### Genome comparison and genetic sequence migration

The tRNA from the chloroplast or nucleus will be involved in the transport of amino acids to reach the number of amino acids required for life. They encode the same 20 amino acids, but *T. sinensis* has one more tRNA than *T.ciliata*. Gene transfer in cells occurs between different organelles, including chloroplasts, mitochondria, and the nucleus [61, 62]. The vast majority of sequences in the mitochondrial genome that migrate from the chloroplast genome are currently considered "dead on arrival", except tRNAs [63]. Expression of chloroplast-derived tRNA genes in the mitochondrial genome has been shown to exist [64, 65]. For the extra tRNA in the *T.sinensis* mitochondria, there is no relationship with the transfer of tRNAs from the chloroplast to the *T. sinensis* mitochondria, which should be associated with the nuclear genome. However, regarding specific gene exchange, it is required to develop in-depth research on gene communication between the nuclear genome and organelles.

### Repeat sequence

Simple sequence repeats (SSRs), also known as microsatellites, are widely distributed on the mitochondrial genome [66]. Due to their high polymorphism and co-dominance, microsatellites are often used as molecular markers to assist in breeding [67] construction of genetic linkage maps and gene mapping, etc. [68].

In contrast, there are some significant variations in SSRs between *T.sinensis* and T.ciliata, for example, *T.ciliata* has one more tetra-nucleotides (CGA), whether this has an evolutionary link to the two plants. It will provide a point for the subsequent screening of genetic molecular markers. In addition, further research on homologous recombination mediated by repeated sequences, *Toona* kinship, and genetic distance will be conducted.

### Systematic evolution

*T.ciliata* and *T.sinensis* have similar morphological characteristics and cultivate in similar environments, so the traditional morphological taxonomy considers the two plants to be cloplantssely related [69, 70]. In the phylogenetic tree, the target tree species *T.ciliata* and *T.sinensis*, belonging to the Meliaceae, clustered into a narrow branch with 100% support. This unifies with the results of traditional morphological taxonomy.

Since plants in the Sapindaceae are more susceptible to geographical location and their genetic variation, the evolutionary distance and genetic variation of plants within the Sapindaceae vary widely [71, 72]. Flora of China records that Meliaceae, Rutaceae, Anacardiaceae, Sapindaceae, and Nitrariacea are natural taxon.

Taxonomists such as Rendle, Hutchinson, and others, who have organ morphological classification, have concluded that Meliaceae and Rutaceae are closely related, but for the classification of the degree of affinity between them, most of them are distinguished from plant physiology and morphology, less from the molecular level of genes [73, 74]. The establishment of the ML evolutionary tree provides a preliminary evolutionary relationship between Meliaceae and Rutaceae at the Mt genome level, but there are limitations because the published Mt genome sequences of plants are still quantitatively insufficient to represent the family level.

## Conclusion

The completion of the Mt genome sequencing of *T.ciliata* and *T. sinensis* has enriched the Mt genome library of *Toona*. and is important for investigating interspecific species relationships and researching the genetics and evolution of *Toona*.

The Mt genomes are predominantly maternally inherited and do not originate in the recombinant genome, therefore, they may have dissimilar evolutionary mechanisms and might reflect different evolutionary information. Further research on gene recombination, locus analysis, etc. can theoretically be supported by the identified moving sequence fragments. Phylogenetic tree building also further illustrates that the simulation of Mt genomic evolutionary tree outcomes is moderately compatible with the traditional classification. The Mt genome can be acclaimed as a molecular marker for the investigative assessment of phylogenetic relationships among species and the genetic structure of populations.

Regarding *T.ciliata* and *T. sinensis*, it is of great value for the data on their energy metabolism, growth and development, and hybrid breeding.

## Supplementary Information

**Additional file 1.**

**Additional file 2.**

**Additional file 3.**

**Additional file 4.**

**Additional file 5.**

**Additional file 6.**

**Additional file 7.**

**Additional file 8.**

**Additional file 9.**

**Additional file 10.**

## Data Availability

The datasets generated during the current study are available in the [NCBI] repository, [*Toona sinensis* (GenBank: OM574631.1) and *Toona ciliata* (GenBank: OM574630.1)].
