# Supplementary material for: Sequencing and analysis of the complete mitochondrial genomes of Toona sinensis and Toona ciliata reveal evolutionary features of Toona
Source: BMC Genomics. 2023 Feb 1;24:58. doi: 10.1186/s12864-023-09150-6 (PMC9893635; doi:10.1186/s12864-023-09150-6)
Supplement: Supplementary file 5 — Additional file 5. [file 12864_2023_9150_MOESM5_ESM.doc]

Species with intact mitochondria in relatives of the target species were downloaded from the NCBI for phylogenetic tree construction. The protein-coding genes of the mitochondrial genomes of the species selected for phylogenetic tree construction were selected separately (generally, the coding genes common to all species were selected for tree construction). MUSCLE v.3.8.31 (http://www.drive5.com/muscle/) software [1] was used to perform the comparison of individual genes among multiple species, and then the genes of each species after comparison were integrated in a certain order in tandem to form the set of protein-coding genes sequences of that species, which were then used for the next step of the analysis. Nucleic acid models were tested using jModelTest 2.1.7 ( https://code.google.com/p/jmodeltest2/) for the selected sequence DNA [2], and the minimum value of AIC (Akaike Information Criterion) was selected as the best model for tree construction.

MrBayes v3.2.7a [3] (http://nbisweden.github.io/MrBayes/manual.html) software, the Bayesian method (BI method: Bayesian Inference) was used to construct the phylogenetic tree.

# Reference

1. Edgar, R.C. (2004) MUSCLE: multiple sequence alignment with high accuracy and high throughput*. Nucleic Acids Res.* **32**(5):1792-1797.
2. David Posada; jModelTest: Phylogenetic Model Averaging, *Molecular Biology and Evolution*, Volume 25, Issue 7, 1 July 2008, Pages 1253–1256.
3. Ronquist, F., M. Teslenko, P. van der Mark, D.L. Ayres, A. Darling, S. Höhna, B. Larget, L. Liu, M.A. Suchard, and J.P. Huelsenbeck. 2012. MRBAYES 3.2: Efficient Bayesian phylogenetic inference and model selection across a large model space. Syst. Biol. 61:539-542.
