# Supplementary material for: Sequencing and analysis of the complete mitochondrial genomes of Toona sinensis and Toona ciliata reveal evolutionary features of Toona
Source: BMC Genomics. 2023 Feb 1;24:58. doi: 10.1186/s12864-023-09150-6 (PMC9893635; doi:10.1186/s12864-023-09150-6)
Supplement: Supplementary file 4 — Additional file 4. [file 12864_2023_9150_MOESM4_ESM.zip › Appendices D/Description of the structure tree.doc]

Species with complete mitochondrial genome sequences and annotations in close relatives of the target species were downloaded from NCBI for phylogenetic tree construction. The protein-coding genes of the mitochondrial genomes of the species selected for phylogenetic tree construction were selected separately (generally, the coding genes common to all species were selected for tree construction). MUSCLE v.3.8.31 (http://www.drive5.com/muscle/) software [1] was used to perform the comparison of individual genes among multiple species, and then the genes of each species after comparison were integrated in a certain order in tandem to form a protein-coding gene sequence set for that species, which was then used for the next step of analysis.jModelTest2.1.7 (https://code.google.com/p/jmodeltest2/) was used to test the selected sequence DNA for nucleic acid model [2], Prottest3.2 (https://code.google.com/p/prottest3/) was used for amino acid model test [3], and AIC (Akaike Information Criterion) The minimum value is the best model for treekeeping. The phylogenetic tree was constructed using RAxML8.1.5 (https://sco.h-its.org/exelixis/web/programming/raxml/index.html) software [4], maximum likelihood method, and the Bootstrap value was set to 1000.

**References**

1. dgar, R.C. (2004) MUSCLE: multiple sequence alignment with high accuracy and high throughput *. Nucleic Acids Res.* **32**(5):1792-1797.
2. David Posada; jModelTest: Phylogenetic Model Averaging, *Molecular Biology and Evolution*, Volume 25, Issue 7, 1 July 2008, Pages 1253–1256.
3. Diego Darriba, Guillermo L. Taboada, Ramón Doallo, David Posada; ProtTest 3: fast selection of best-fit models of protein evolution, *Bioinformatics*, Volume 27, Issue 8, 15 April 2011, Pages 1164–1165.
4. Stamatakis A. RAxML version 8: a tool for phylogenetic analysis and post-analysis of large phylogenies. Bioinformatics. 2014 May 1;30(9):1312-3. doi: 10.1093/bioinformatics/btu033. Epub 2014 Jan 21. PMID: 24451623; PMCID: PMC3998144.
